# Simple, Inexpensive Technique for High-Quality Smartphone Fundus Photography in Human and Animal Eyes

**DOI:** 10.1155/2013/518479

**Published:** 2013-09-19

**Authors:** Luis J. Haddock, David Y. Kim, Shizuo Mukai

**Affiliations:** Retina Service, Massachusetts Eye and Ear Infirmary, and Department of Ophthalmology, Harvard Medical School, 243 Charles Street, Boston, MA 02114, USA

## Abstract

*Purpose*. We describe in detail a relatively simple technique of fundus photography in human and rabbit eyes using a smartphone, an inexpensive app for the smartphone, and instruments that are readily available in an ophthalmic practice. *Methods*. Fundus images were captured with a smartphone and a 20D lens with or without a Koeppe lens. By using the coaxial light source of the phone, this system works as an indirect ophthalmoscope that creates a digital image of the fundus. The application whose software allows for independent control of focus, exposure, and light intensity during video filming was used. With this app, we recorded high-definition videos of the fundus and subsequently extracted high-quality, still images from the video clip. *Results*. The described technique of smartphone fundus photography was able to capture excellent high-quality fundus images in both children under anesthesia and in awake adults. Excellent images were acquired with the 20D lens alone in the clinic, and the addition of the Koeppe lens in the operating room resulted in the best quality images. Successful photodocumentation of rabbit fundus was achieved in control and experimental eyes. *Conclusion*. The currently described system was able to take consistently high-quality fundus photographs in patients and in animals using readily available instruments that are portable with simple power sources. It is relatively simple to master, is relatively inexpensive, and can take advantage of the expanding mobile-telephone networks for telemedicine.

## 1. Introduction

The ever increasing popularity and availability of smartphones and the rapid advances in technology for capturing and sharing images with them have resulted in the expanding use of smartphones as a clinical-imaging device in ophthalmology. This application has been facilitated by the ease of use and portability of the smartphones and the already extensive mobile-phone networks, and it presents a unique opportunity for applications such as telemedicine and self-diagnosis [1].

Retinal photography (fundus photography) is an essential part of ophthalmology practice. Acquisition of high-quality fundus images requires a combination of appropriate optics and illumination usually in the form of a condensing lens and a coaxial light source [2]. This is part of the reason that a commercial fundus camera normally costs tens to hundreds of thousand dollars.

We describe in detail a relatively simple technique of fundus photography in human and rabbit eyes using a smartphone, an inexpensive app for the smartphone, and instruments that are readily available in an ophthalmic practice. The safety of the illumination using this technique with an iPhone 4 in human eyes has been described previously [3].

## 2. Methods

### 2.1. Technique

**Figure 1:**
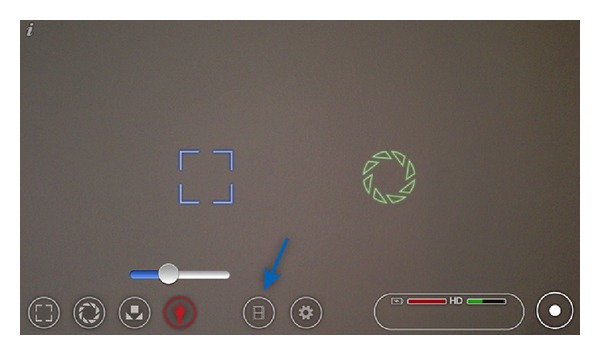
Filmic Pro app allows independent control of light intensity (red light bulb), exposure (green circle), and focus (blue square) while filming. Video library access (blue arrow).

Smartphone fundus images were captured with an iPhone 4 or iPhone 5 (Apple Inc., Cupertino, CA, USA) and a 20D lens (Volk Optical Inc., Mentor, OH, USA) with or without a Koeppe lens (Ocular Instruments, Bellevue, WA, USA). By using the coaxial light source of the phone, this system worked as an indirect ophthalmoscope that captured a digital image of the fundus in the smartphone camera [2]. The application (app) Filmic Pro (Cinegenix LLC, Seattle, WA, USA; http://filmicpro.com/) (Figure 1) was used because its software allowed for independent control of the focus, exposure, and light intensity during video filming. With this app, high-definition videos of the fundus were recorded for subsequent high-quality, still-image extraction. Without this app, it was extremely difficult to obtain good quality fundus images.

**Figure 2:**
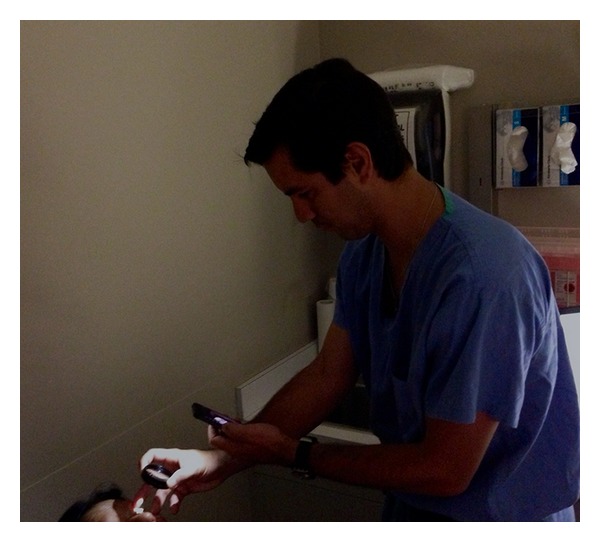
Filming setup with user holding iPhone for filming with Filmic Pro app in one hand and holding 20D lens for focusing on the retina in the other hand.

This technique of smartphone fundus photography involved multiple steps that are described in detail below. The technique is simple, yet it may take a few attempts to master since the user must learn to readjust the filming distance for focusing with the 20D lens while looking into an inverted image of the fundus on the iPhone screen. In addition, since the camera lens is usually located in the corner of the smartphone and the digital display is in the center of the phone, the parallel but skewed alignment necessitated by this displacement required some practice to get the fundus images in the center of the screen (Figure 2). Good pupillary dilation prior to filming was ideal as this allowed for easier imaging of the fundus. Before filming, the Filmic Pro app had to be configured to allow for proper control of light intensity, exposure, and focus. In the latest update of this app (Filmic Pro V 3.2), independent control of the iPhone light intensity was available, and for best exposure, the light intensity was set to its lowest level (Figure 1—light bulb). When using the older versions of the app in which there was no independent control of light intensity, we placed a strip of Micropore paper medical tape (3 M, St. Paul, MN, USA) over the light source in the front of the phone to reduce the light intensity adequately for successful image acquisition. The exposure lock was set to “off” since independent control of the exposure was needed in order to toggle the exposure control to the desired area of the fundus to be imaged (Figure 1—green circle). Finally, the focus lock was set to “off” in order to maintain independent control of the camera's focus during the imaging of the desired area of the fundus as it appeared on the screen (Figure 1— blue square).

Once the app was set to the above parameters, the iPhone light source and camera were used by the operator as an indirect ophthalmoscope to create a digital image. For most images, we used a 20D lens for focusing the light on the patient's retina in the clinic or emergency room setting. In the operating room during examinations under anesthesia, we also used a combination of a 20D lens with a Koeppe lens, which when placed on the eye was useful in keeping the lids open, the cornea wet, and provided a slightly wider field of view. The app's video recording was activated, and a video of the fundus was captured on the iPhone screen (Figure 2). The exposure and focus controls were toggled to the desired area of the fundus on the screen with the filming hand until the fundus was filmed successfully. Since this has to be done with one hand (the other hand is holding the 20D lens), we found that holding the smartphone vertically with the camera lens up and using the touch screen with the thumb worked the best (Figure 2). Once the areas of interest were filmed, the video library of the app was accessed (Figure 1—arrow), and the recorded video was selected and exported to the camera roll.

After the video had been exported to the camera roll, the still images were extracted by one of two methods. The first method involved the use of either the app MovieToImage (DreamOnline, Inc., Tokyo, Japan; http://www.dreamonline.co.jp/) or Video 2 Photo (Francis Bonnin, PacoLabs; http://pacolabs.com/) that allowed the video clip still-image extraction. The alternative method involved playing the video and pausing at the desired image in order to take a screenshot (click and hold the top power button and the round menu button simultaneously) of the desired video image. Once the still images were exported to the camera roll by using either method, the images were edited by using the iPhone's editing function or the desired photo editing app in order to crop, rotate, and enhance the fundus photos. Using the apps that allowed still-image extraction resulted in better image quality and easier image selection. Finally, the high-quality images from the patients were ready to be archived in the medical record or used for telemedicine.

It was of utmost importance to maintain the confidentiality of personal data in accordance with the Data Protection Act 1998 and Access to Health Records Act 1990. For protection of privacy, the smartphones used in our institution are encrypted, and the images are transmitted via institutional e-mail with encryption of the attachments.

## 3. Results

**Figure 3:**
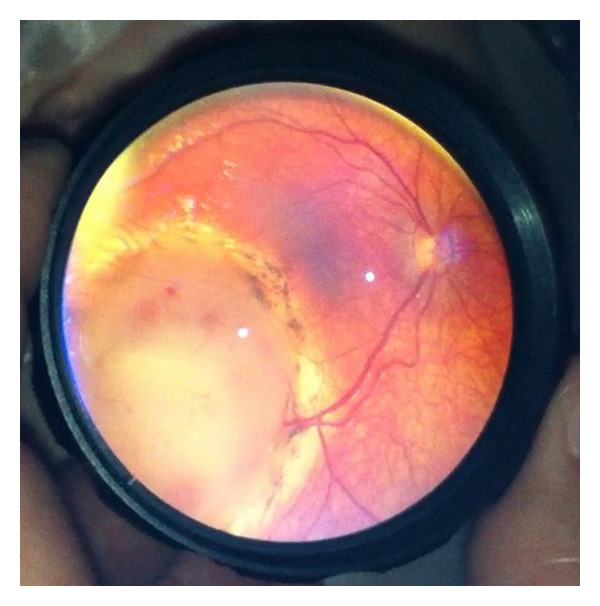
Retinoblastoma (partially treated) imaged during examination under anesthesia.

**Figure 4:**
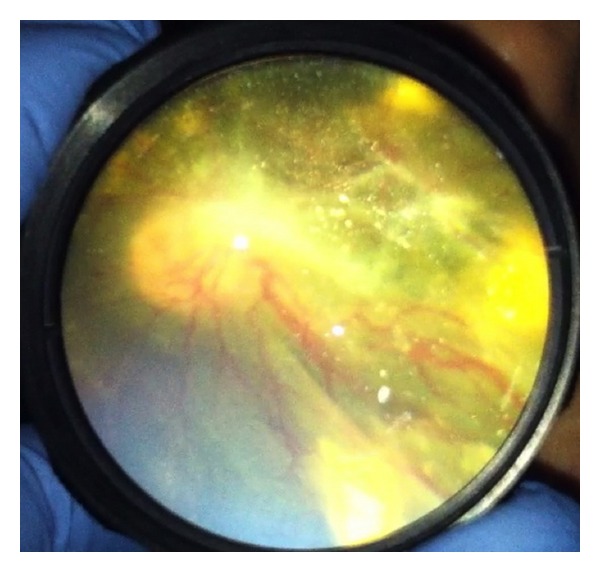
Familial exudative vitreoretinopathy imaged during examination under anesthesia.

**Figure 5:**
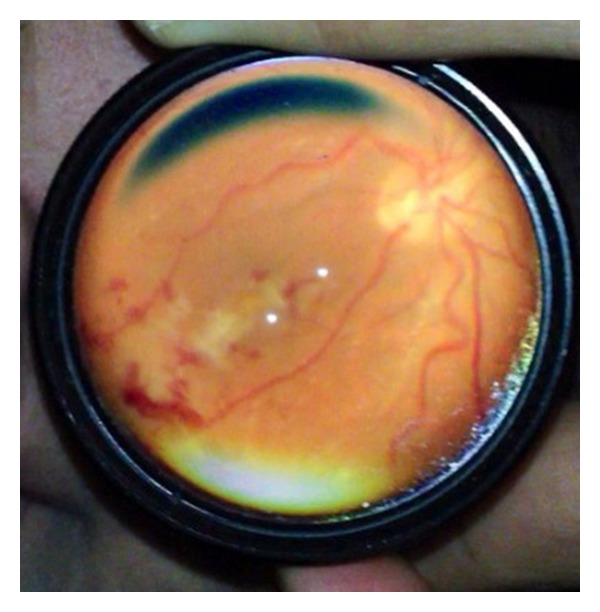
Vasculitis imaged in emergency department setting.

**Figure 6:**
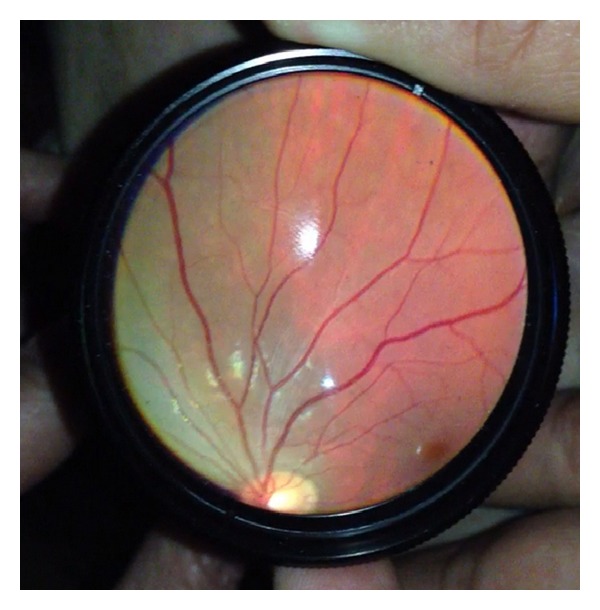
Large choroidal nevus imaged in the emergency department setting.

The described technique of smartphone fundus photography with the use of iPhone 4 or iPhone 5, the app Filmic pro, and a 20D lens with or without a Koeppe lens was able to capture excellent, high-quality fundus images in both children under anesthesia (Figures 3 and 4) and in awake adults (Figures 5 and 6). The best results were achieved in the operating room when a Koeppe lens was used in addition to the 20D lens; however, excellent images were acquired with the 20D lens alone in the clinic and emergency room setting as well as in the operating room. We found that even first-year ophthalmology residents were able to master this technique in relatively short period.

**Figure 7:**
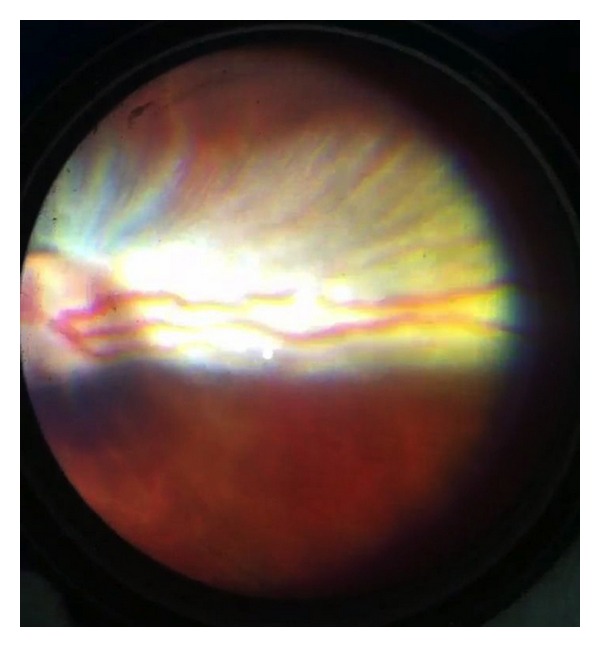
Control rabbit eye.

**Figure 8:**
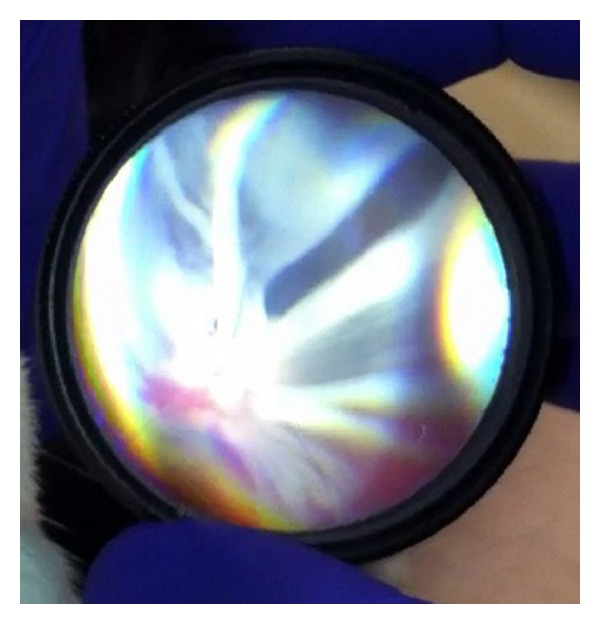
Experimental rabbit eye with induced total retinal detachment and severe proliferative vitreoretinopathy.

In addition, successful photodocumentation of rabbit fundus was achieved in control and experimental eyes (Figures 7 and 8). Because of the smaller eye, a 28 D or 30 D lens worked better than a 20D lens in some instances.

## 4. Discussion

High-quality fundus images were successfully captured in human and rabbit eyes using the built-in camera and light source of the iPhone 4 and iPhone 5 in combination with the application Filmic Pro and a 20D lens with or without a Koeppe lens. This technique produced consistently high-quality images because it allowed independent control of the light intensity, the focus, and the exposure during filming. In addition, with the use of the video capture mode with subsequent still-image extraction, high-quality images were obtained even with a relatively short time of video capture, as the best available still images were extracted subsequently. We found that using the combination of the app with video capture was critical to the success of our technique.

The iPhone 4 light source, when used with a 20D condensing lens for smartphone fundus photography using the described technique, had been previously tested and found to be well within the safety standards for human eyes. Kim et al. [3] showed that the light levels (without decrease using the app or the paper tape) were 150 times below the thermal hazard limit and 240 times below the photochemical hazard limit set by the International Organization for Standardization (ISO 15004-2.2) [4, 5] and 10 times below the levels produced by the commercially available Keeler Vantage Plus LED indirect ophthalmoscope. In addition, as described in the Techniques section under Methods, we always used the lowest intensity level using the app or placed paper tape over the light source to significantly reduce the light for video recording. Therefore, we were working at a level of light intensity and energy well below what was measured in the Kim et al. paper. Although we did not measure the light intensity and energy levels of the iPhone 5, even if the light source was significantly higher than that of iPhone 4, we felt that the range of illumination in which we were recording was safe. Nonetheless, the light source of iPhone 5 and other smartphones need to be measured to verify safety.

Smartphones are now being used more routinely in ophthalmology to document patient's ocular conditions [6, 7]. Previously described techniques of fundus imaging were found to be often difficult to repeat [2, 6]. This was partly because video capture with Apple's built-in camera app in the iPhones proved to be challenging due to its inability to independently control the focus and the exposure during filming thereby resulting in glare and poor image quality. The described technique provided a relatively simpler and higher quality way to more consistently produce excellent images of a patient's fundus.

This technique has been extremely helpful for us in the emergency department setting, in inpatient consultations, and during examinations under anesthesia as it provided a cheaper and portable option for high-quality fundus-image acquisition for documentation and consultation. This technique is well tolerated in awake patients most likely since the light intensity used is often well below that used in standard indirect ophthalmoscopy.

In addition, this technique has been useful in the laboratory to document retinal findings *in vivo* in rabbits. Since one of the authors (Shizuo Mukai) has been successful in photographing the retina of smaller animals including mice using a conceptually similar system using the Kowa RC-2 fundus camera (Kowa Optimed Inc., Torrance, CA, USA) instead of the iPhone 4 or iPhone 5 and 90 D, 78 D, or 60 D lens (Volk Optical Inc., Mentor, OH, USA) instead of a 20D lens [8], we predict that the currently described technique can be modified for use in smaller animals including mice.

## 5. Conclusion

The currently described system was able to take consistently high-quality fundus photographs in patients and in animals using readily available instruments that are portable with simple power sources. It is relatively simple to master, is relatively inexpensive, and can take advantage of the expanding mobile-telephone networks for telemedicine. We expect that the quality of the images achieved using this technique will continue to improve with availability of higher-resolution cameras with larger sensors and better image stabilization that are being incorporated into newer smartphones.

## References

[1] Lamirel C, Bruce BB, Wright DW, Newman NJ, Biousse V (2012). Nonmydriatic digital ocular fundus photography on the iPhone 3G: the FOTO-ED study. *Archives of Ophthalmology*.

[2] Bastawrous A (2012). Smartphone fundoscopy. *Ophthalmology*.

[3] Kim DY, Delori F, Mukai S (2012). Smartphone photography safety. *Ophthalmology*.

[4] International Standard, ISO, 15004-2.2 (2007). *Ophthalmic Instruments, Light Hazard Protection*.

[5] Sliney DH, Mellerio J, Gabel V, Schulmeister K (2002). What is the meaning of threshold in laser injury experiments? Implications for human exposure limits. *Health Physics*.

[6] Lord RK, Shah VA, San-Filippo AN, Krishna R (2010). Novel uses of smartphones in ophthalmology. *Ophthalmology*.

[7] Chhablani J, Kaja S, Shah V (2012). Smartphones in ophthalmology. *Indian Journal of Ophthalmology*.

[8] MacPherson D, Conkrite K, Tam M, Mukai S, Mu D, Jacks T (2007). Murine bilateral retinoblastoma exhibiting rapid-onset, metastatic progression and N-myc gene amplification. *The EMBO Journal*.

